# The behavioral phenotype of children and adolescents with attenuated non-ketotic hyperglycinemia, intermediate to good subtype

**DOI:** 10.1186/s13023-024-03172-3

**Published:** 2024-04-08

**Authors:** Liesbet D. F. M. Van Hirtum, Tine Van Damme, Johan L. K. Van Hove, Jean G. Steyaert

**Affiliations:** 1 Department of Child- and Adolescent Psychiatry, KU Leuven, Leuven, Belgium; 2 Ankerpunt vzw, Leuven, Belgium; 3 Department of Child Psychiatry, UPC Z.Org, Leuven, Belgium; 4 Department of Rehabilitation Sciences, KU Leuven, Leuven, Belgium; 5 Section of Clinical Genetics and Metabolism, Department of Pediatrics, University of Colorado, Aurora, Colorado USA; 6 Leuven Autism Research Consortium, Leuven, Belgium

**Keywords:** Non-ketotic hyperglycinemia, Developmental delay, Hyperactivity, Behavior, Disruptive, Dextromethorphan

## Abstract

**Aim:**

We aim to describe the behavioral phenotype of children and adolescents with the good to intermediate attenuated form of non-ketotic hyperglycinemia (NKH) and to explore associations between the behavioral phenotype and age, sex, plasma glycine levels and drug treatment.

**Method:**

Parents of children with attenuated NKH completed questionnaires assessing maladaptive behavior, adaptive behavior, social communication, speech/language development and motor development in addition to demographic and medical questions.

**Results and interpretation:**

Twelve children, age 6 to 21y, functioned at mild to severe intellectual disability levels. Their speech/language development was in line with their developmental quotient. Relative to their intellectual functioning, their motor development and communication were weaker in comparison to their general development. Their adaptive behavior, however, appeared a relative strength. There was no evidence for autism spectrum disorder occurring more frequently than expected, rather social skills, except for communication, were rated as a relative strength. Maladaptive behaviors with ADHD-like characteristics were present in more than two thirds of children. Maladaptive behaviors were significantly related to female sex and to taking dextromethorphan, but no significant relation between plasma glycine levels and behavior was found. Future studies will need to evaluate causality in the observed relation between dextromethorphan use and maladaptive behaviors. Clinicians should reconsider the benefit of dextromethorphan when presented with disruptive behaviors in children with attenuated NKH.

## Introduction

Non-ketotic hyperglycinemia (NKH) is an ultra-rare (incidence 1/76,000 births) genetic, neurometabolic disorder caused by deficient enzyme activity of the glycine cleavage enzyme (GCE) (EC 1.4.4.2) due to mutations in *GLDC* encoding the P-protein or *AMT* encoding the T-protein. The GCE is the main catabolic enzyme of glycine and its deficiency results in elevated levels of the amino acid glycine in different tissues including the brain [1, 2].

The most common phenotype associated with NKH is the severe form of classic NKH where patients lack developmental progress, have spasticity and therapy resistant epilepsy (Table 1) [2–4]. The attenuated phenotypic form of NKH occurs in one out of six children with NKH [2–5]. Children with the attenuated form have no or treatable epilepsy and make developmental progress, which phenotypically is divided into three categories: poor (Developmental Quotient (DQ)<20), intermediate (DQ 20-50), and good (DQ>50) (Table 1) [2, 4]. The severe neurological dysfunction and profound intellectual disability (ID) impede differentiation of behavioral problems in severe or attenuated poor NKH. Patients with a good to intermediate form of attenuated NKH have a substantially better prognosis [4]. However, parents of these children frequently encounter marked behavioral problems, which by parental report are poorly or not responsive to usual medication approaches [6–12].

**Table 1:** The different types of non-ketotic hyperglycinemia

**Forms of NKH**		**Criteria**
* Classic NKH		Primary GCE-deficiencies due to pathogenic variants in one of the GCE protein components, the P protein (*GLDC* gene) or the T protein (*AMT* gene)
1) Severe NKH		- Neonatal epileptic encephalopathy and transient coma for two to three weeks - Minimal developmental progress - Spasticity - Therapy-resistant epilepsy
2) Attenuated NKH		It may present in the neonatal period with a similar transient comatose episode, but may also present later during infancy.
	a) Poor	- Children usually suffer from epilepsy - Very limited development (DQ < 20) - Only sitting or at best only walking a few steps - No or very limited speech
	b) Intermediate	- Marked development (DQ 20-50) - No or treatable epilepsy - Frequently encounter marked behavioral problems
	c) Good	- Marked development (DQ >50) - No or treatable epilepsy - Frequently encounter marked behavioral problems
* Variant NKH		Caused by pathogenic variants in other genes with an effect on GCE

*Abbreviations*: *NKH* Non-ketotic hyperglycinemia, *GCE* Glycine Cleavage Enzyme, *DQ* Developmental quotient

Current treatment consists of glycine reduction and mitigation of purported glycine toxicity. There are two glycine-reduction strategies aimed at normalizing plasma glycine levels to 120-300 µM [2], which reduces but does not normalize brain glycine levels [13]. First, benzoate is given, which conjugates with glycine and is excreted in the urine as hippurate thus lowering plasma glycine levels [3, 14, 15]. Second, ketogenic diet results in glycine lowering by use of glycine as a gluconeogenic precursor [13]. Both glycine lowering treatments result in increased alertness and improved seizure control, but the effect on behavior is not reported. One hypothesis of the purported neurotoxicity of elevated glycine in pathogenesis of NKH is based on overstimulation of the N-methyl-D-aspartate (NMDA) type of glutamatergic receptors [12, 16, 17]. As treatment of hypothetical NMDA receptor overstimulation, dextromethorphan (DMP) in high doses [16, 18], or ketamine [19], have been used as partial NMDA-receptor antagonists. For patients with attenuated NKH, the combination of treatment with both benzoate and DMP has been reported to contribute to a better developmental outcome if started early [20].

For patients with attenuated NKH, treating physicians mention hyperactivity and problems in attention and concentration as a major problem. Further, a few cases of intermediate attenuated NKH were recognized in series of genetic studies in children with autism [11]. Yet, most articles mention behavioral problems without formal studies. A study of the behavioral phenotype in attenuated NKH is lacking, and the relationship between the behavioral phenotype and key features of the disorder (such as genetic profile, glycine plasma levels and drug treatment) remains to be investigated.

The present exploratory study charts the behavioral phenotype of attenuated NKH patients using standardized questionnaires. The study was limited to the good and intermediate form of attenuated NKH. In moderate and severe ID, a dimensional approach is most appropriate to support Diagnostic and Statistical Manual of Mental Disorders (DSM-5) classifications. We aim to explore associations between the dimensions in the behavioral phenotype and age, biological sex, glycine plasma levels and drug treatment.

## Methods

### Ethics approval

This study was approved by an independent ethics committee in Leuven, Belgium, named EC Research UZ/KU Leuven with study number MP017713. Patients were also further enrolled in the IRB-approved study in Colorado by the ethics committee named Colorado Multiple Institutional Review Board with number COMIRB# 05-0790. Participation was initiated after obtaining informed consent, which included consent for publication. All procedures were done in accordance with the ethical standards of the ethics committee in accordance with the Helsinki Declaration of 1975 and as revised in 2000. Participation was initiated after obtaining informed consent.

### Procedure

Parents of children with NKH were invited to participate through an information letter, through patient association groups (NKH Crusaders, Maud & Vic Foundation), by treating physicians, or by recontacting participants of the Colorado study "Prognosis in nonketotic hyperglycinemia”.

Inclusion criteria included (a) a diagnosis of classic NKH documented by genetic testing in the child [2]; (b) chronological age between 6 and 21 years old; (c) sufficient knowledge of the Dutch or English language of the parents or caregivers to understand and complete the questionnaires; (d) ability to walk without support; (e) ability to use at least one word or 10 signs for communication, and (f) treatment with no more than one anti-epileptic medication. Cases of variant NKH or another genetic cause were excluded [21]. Data were collected via the online platform REDCap (Research Electronic Data Capture), hosted on a server at KU Leuven [22, 23]. The record identification numbers shown in the tables come from the pseudonymization by REDCap.

The parents were invited to complete the questionnaires electronically. Following questions on the inclusion criteria, additional demographic and medical questions were compiled on age, biological sex, presence of epilepsy, current medication use, and the most recent plasma glycine level. The genetic information, which had been obtained as part of medical care, was captured in the Colorado study. Parents further uploaded available intelligence quotient (IQ) reports or reports for educational support.

Intellectual functioning was subdivided according to the International Classification of Diseases (ICD)-10 [24], which classified intellectual functioning as severe ID with IQ 20-34 corresponding to a developmental age of 3-5 y for adults, moderate ID with IQ 35-49 corresponding to a developmental age of 6-9 y, and mild ID with IQ 50-70 corresponding to developmental age of 9-12 y, whereas an IQ 70-85 is considered weakly gifted.

### Survey instruments

The parents or caregivers completed seven questionnaires described below using language (English or Dutch) and age-appropriate versions of the questionnaires and language and region-appropriate norms. If local norms were unavailable, we recognized that such differences are generally small within this population of cognitively challenged children. Clinically relevant parental comments were extracted from free comments.

The Developmental Behavior Checklist parent form (DBC-2-P) is a questionnaire to assess emotional and behavioral problems of children with intellectual disabilities between ages 6 and 18 y. Using 95 behavioral descriptions that might apply to the child over the past six months, a Total Problem Score (TPS) and five problem subscales were derived: Disruptive, Self-absorbed, Communication disturbance, Anxiety, and Social relating. We considered a score that falls in the highest quartile as problematic [25, 26].

The Adaptive Behavior Assessment System (ABAS-3 5-21 y) [27, 28] evaluates adaptive behavior in children between 5 and 21 y of age. Scores on different domains of adaptive function (Community use, Communication, Functional academics, Home and school living, Leisure, Health and safety, Self-direction, Self-care, and Social) combine into a General Adaptive Composite (GAC). A score <-1 Standard Deviation (SD) is considered low, and a score <-2SD (norm score <70) as very low and a limitation in adaptive behavior [28]. Flemish norm scores are defined down to a score of 55 [27], whereas the US version includes lower norm scores [28].

The Social Communication Questionnaire (SCQ) 'Lifetime’ version [29, 30] assesses autism spectrum disorder (ASD) characteristics, and suggests possible ASD above a summed score of 15. The SCQ can be used up from 4 y of age and older and is reliable for individuals with mild to moderate ID [29, 30]. In addition to questions related to when the child was age 4 to 5 y, given the marked developmental delays of these children, we decided to also provide questions based on behaviors of the child at its current age.

The Children's Communication Checklist (CCC-2) screens for difficulties in communication skills [31, 32], with a focus on identifying pragmatic language impairments, which may be associated with ASD. It is designed for children between ages 4 y and 15.5 y, who speak at least in two words sentences. The 70 question survey is divided into four scales on language structure (Speech, Syntax, Semantics, and Coherence), four scales on pragmatic aspects of communication (Inappropriate Initiation, Stereotyped Language, Use of Context, and Nonverbal Communication), and two scales on autism related measures (Social Relationships and Interests), which are combined in a General Communication Score (GCC), a pragmatic score, and a Social Interaction Score (SIDI). Scores ≥90^th^ percentile are rated as weak, and ≥98-99^th^ percentile as very weak, and score ≥95^th^ percentile on two or more scales indicate significant communication problems. An elevated GCC combined with a low SIDI score (<10^th^ percentile) indicate structural language problems and, combined with a high SIDI (>90^th^ percentile), indicate pragmatic problems. A high GCC with either a high SIDI or a high Social Relationships and Interests scale may indicate ASD [31, 32].

For assessment of motor development and motor skills, three different questionnaires were used for all children, in order to cover the full range of developmental ages of the participants. The motor skill checklist (MSC) for toddlers is applicable for ages 3-5 y [33], the Developmental Coordination Disorder Daily Questionnaire (DCDDaily-Q) for ages 5-8 y [34], and the checklist of the Movement Assessment Battery for children (M-ABC-2-Checklist) for ages 5-12 y [35, 36]. This also covers the average age equivalents in adults for severe, moderate and mild ID respectively [24].

The MSC questionnaire targets motor performance in daily activities and was developed in Dutch as “*Vragenlijst voor de Motorische Vaardigheden van Kleuters*” (VMVK), of which we made a back-to-back translation into English (available upon request from the authors). The motor development age to which the scores of the participating children corresponded was determined.

The DCDDaily-Q is a parent reported questionnaire addressing the children’s performance in Activities of Daily Living (ADL), evaluated for quality of performance, frequency of participation, and whether it took them longer to learn the skill. Scores were age-matched for functionality. We compared the child’s score obtained with those of children aged 5 y (the lower limit of this questionnaire), unless the child achieved a score appropriate to a higher developmental age (indicated in Table 2) [34].

**Table 2:** Summary of the results of the behavioral, cognitive, communication, and motor related questionnaires

Study number		16	17	19	23	28	34	44	45	49	70	71	73
Biological sex		Female	Male	Female	Male	Male	Female	Male	Female	Male	Female	Female	Male
DBC	TPS	68 (P75-90)	15 (< P50)	52 (P75-90)	47 (P50-75)	58 (P75-90)	40 (P50-75)	61 (P75-90)	83 (P90-95)	31 (P50-75)	41 (P50-75)	57 (P75-90)	54 (P75)
	Disruptive	24 (P75-90)	3 (< P50)	22 (P75-90)	18 (P50-75)	22 (P75-90)	16 (P50-75)	30 (> P95)	31 (P95)	7 (< P50)	14 (P50-75)	18 (P50-75)	12 (< P50)
	Self-absorbed	23 (P90-95)	5 (< P50)	13 (P50-75)	15 (P50-75)	21 (P90-95)	3 (< P50)	13 (P50-75)	30 (> P95)	9 (P75)	20 (P75-90)	20 (P75-90)	28 (> P95)
	Comm. Dist.	10 (P90)	2 (< P50)	9 (P75-90)	7 (P50-75)	8 (P75)	6 (P50-75)	7 (P50-75)	8 (P75)	5 (P75)	4 (< P50)	6 (P50-75)	4 (< P50)
	Anxiety	7 (P50-75)	2 (< P50)	6 (P75)	4 (P50)	4 (< P50)	9 (P75-90)	6 (P50-75)	11 (P95)	5 (P75-90)	0 (< P50)	6 (P50-75)	4 (< P50)
	Social relating	2 (< P50)	0 (< P50)	4 (P50-75)	3 (P50)	1 (< P50)	6 (P50-75)	3 (< P50)	3 (P50-75)	3 (P50-75)	5 (P50-75)	3 (< P50)	5 (P50-75)
SCQ	Total score	17	11	25	22	19	7	27	20	13	13	14	15
	Current	10	6	15	7	14	7	15	9	13	10	11	14
ABAS	GAC	61 (P0.5 – EL)	61 (P0.5 – EL)	< 55 (< P0.1 –EL)	56 (P0.2 – EL)	56 (P0.2 – EL)	76 (P5 – L)	67 (P1 – EL)	61 (P0.5 – EL)	62 (P0.6 – EL)	47 (< P0.1 –EL)	60 (P0.4 – EL)	47 (< P0.1–EL)
	Conceptual	57 (P0.2 – EL)	57 (P0.2 – EL)	< 55 (< P0.1 –EL)	< 55 (< P0.1 –EL)	56 (P0.2 – EL)	73 (P4 – L)	69 (P2 – EL)	62 (P0.6 – EL)	55 (< P0.1 –EL)	49 (< P0.1 –EL)	62 (P1 – EL)	49 (< P0.1–EL)
	Practical	56 (P0.2 – EL)	60 (P0.4 – EL)	58 (P0.3 – EL)	64 (P0.8 – EL)	68 (P2 – EL)	82 (P12 – BA)	64 (P1 – EL)	60 (P0.4 – EL)	67 (P1.4 – EL)	48 (< P0.1 –EL)	59 (P0.3 – EL)	48 (< P0.1–EL)
	Social	86 (P18 – BA)	83 (P12.9 – L)	57 (P0.2 – EL)	57 (P0.2 – EL)	56 (P0.2 – EL)	79 (P8 – L)	77 (P6 – L)	77 (P6.3 – L)	75 (P4.8 – L)	54 (P0.1 –EL)	70 (P2 – EL)	54 (P0.1–EL)
CCC-2	Scales ≥ P95 (n)	7/10	4/10	10/10	///	7/10	3/10	9/10	5/10	Non-valid	///	7/10	///
	GCC	> P99	> P99	> P99		> P99	P90-95	> P99	P95-98			> P99	
	Pragm. Score	P98	P90-95	> P99		P95-98	P80	P99	P90-95			P95-98	
	SIDI	P2-5	P2	P15-20		P2-5	P2-5	P30	P15			P10-15	
	Soc.Rel scale	Average	Average	Very weak		Average	Average	Average	Weak			Average	
MSC	Total	87	36	64	71	46	41	50	90	47	69	68	109
	Age equivalent	< 36m	65-66m	41-42m	38m	54m	59m	51m	< 36m	53m	38-39m	39m	< 36m
DCDDaily (i.e.5y)	Participation	68 (≥ P95)	31 (< P85 ~7-8y)	63 (≥ P95)	55 (≥ P95)	42 (< P85 ~6y)	45 (< P85)	36 (< P85 ~7-8y)	44 (< P85)	41 (< P85~6y)	67 (≥ P95)	52 (≥ P95)	Non-valid
	Quality	63 (≥ P95)	31 (< P85 ~7-8y)	52 (≥ P95)	58 (≥ P95)	48 (≥ P95)	39 (P85-95~5-6y)	44 (≥ P95)	65 (≥ P95)	35 (< P85 ~6y)	56 (≥ P95)	57 (≥ P95)	
	Acquisition	≥ P95	≥ P95	≥ P95	≥ P95	≥ P95	≥ P95	≥ P95	≥ P95	≥ P95	≥ P95	≥ P95	
M-ABC	TMS (age)	50 (< 5y)	9 (5y)	34 (< 5y)	37 (< 5y)	19 (6y)	21 (5y)	21 (5y)	60 (< 5y)	16 (5y)	32 (5y)	53 (< 5y)	Non-valid
	Section C (n/13)	7	5	5	4	8	11	10	4	10	5	10	6
	Probl acc parent	Yes	Yes	No	Yes	No	No	Yes	Yes	Yes	No	Yes	Yes
ADHD	~ DBC (n/9)	8	4	8	7	7	5	7	9	6	8	8	6
	~M-ABC (n/5)	5	1	4	3	5	5	4	3	4	4	4	4

*Abbreviations*: *TPS* Total Problem Score, *P* Percentile, *Comm. Dist.* Communication disturbance, *GAC* General Adaptive Composite, *EL* Extremely low, *L* Low, *BA* Below average, *n N*umber, *GCC* General Communication Composite, *Pragm. Score* Pragmatic score, *SIDI* Social Interaction Difference Index, *m* month, *y* year, *TMS* Total Motor Score, *Probl. Acc. Parent* Problem in motor functioning according to parents

The M-ABC-2 Checklist assesses whether and how well a child can perform certain movements in either a static and predictable environment (section A), or in a dynamic or unpredictable environment (section B), and are combined into a Total Motor Score. A higher score reflects more motor difficulties. The motor development age with which the scores of the participating children corresponded was also determined. Questions in Section C that identify non-motor factors that can influence a child's ability to learn and perform motor skills, were assessed qualitatively [35, 36].

There are no appropriate instruments available adapted for children with ID to evaluate ADHD [37, 38], a frequently reported concern in NKH children [6–10, 39, 40]. Given the collected data, we used DBC and M-ABC-2 items reflecting DSM-5 symptoms of attention deficit hyperactivity disorder (ADHD) as a proxy (See Table 3). This yields nine unique items corresponding to nine of the 18 ADHD symptoms in the DSM-5. We set an arbitrary cut-off of six out of nine items for a provisional diagnosis of ADHD (a similar proportion as 12/18 criteria required for a diagnosis of ADHD-combined type in DSM-5 [25, 26, 35, 36, 39]).

**Table 3:** Attention deficit hyperactivity disorder characteristics

*Items in the DBC corresponding to ADHD characteristics* [25, 26]
9. Cannot attend to one activity for any length of time. Has poor attention span.
19. Is easily distracted from tasks (e.g., by noises).
35. Is impatient.
37. Is impulsive, acts before thinking.
49. Is noisy or boisterous.
50. Is very active or restless. Can't sit still.
55. Has poor sense of danger.
81. Talks too much or too fast.
84. Has unconnected thoughts. Different ideas are jumbled together with unclear meaning.
*Items part C of M-ABC-Checklist section C corresponding to ADHD subdomains*
C.1 Disorganized (e.g. scattered clothes slows up dressing after PE; puts on shoes before socks)
C.2 Hesitant/forgetful (e.g. slow to start complex actions; forgets what to do in the middle of an action sequence)
C.6 Impulsive (e.g. starts before instructions are complete; impatient of detail)
C.7 Distractible (e.g. looks around; responds to irrelevant noises)
C.8 Overactive (e.g. squirms and fidgets; movers constantly when listening to instructions, fiddles with clothes)

### Statistical analysis

Normality of distribution was first evaluated using both the Shapiro-Wilk and Kolmogorov-Smirnov statistics. Descriptive statistics for normally distributed data were described by the mean and standard deviation, and for data that are not normally distributed by the median, the interquartile range, and the range. For comparison between two conditions, either the Student t-test or the Mann-Whitney-U test was performed, depending on normality of data distribution or not, respectively. Correlations between two variables were evaluated by the Pearson or Spearman rank correlation, depending on the normality of distribution or not, respectively.

For normally distributed variables, the interacting effect of two parameters on the outcome variable was evaluated using an ANCOVA statistic in a linear model. Bonferroni correction was used for multiple comparisons. Statistical analysis was done using SPSS vs. 28.0.1.1 (IBM, Armonk, NY).

## Results

### Population

The parents of 12 children participated, of which seven with an English-speaking background and five with other backgrounds. 10 children had mutations in *GLDC* and 2 in *AMT,* and all carried at least one missense variant, the majority of which is known to confer residual activity (Table 4). There were equal numbers by sex, presence of epilepsy or not, and taking DMP or not (Table 5). All children used benzoate, but only five children achieved target plasma glycine levels [2]. Four children received psychopharmaceuticals of several groups.

**Table 4:** Genetic variants in enrolled individuals

Study number	Gene	DNA1	Protein1	DNA2	Protein2	Alleles with residual activity
16	*GLDC*	Del exons 16-26	absent	c.605C>T	p.Ala202Val	1
17	*AMT*	c.317C>T	p.Ile106Thr	c.959G>A	p.Arg320His	1
19	*GLDC*	c.1055C>G	p.Thr352Arg	c.2405C>T	p.Ala802Val	1
23	*AMT*	c.317T>C	p.Ile106Thr	c.317T>C	p.Ile106Thr	2
28	*GLDC*	c.2714T>C	p.Val905Gly	c.2183G>A	p.Gly728Glu	1
34	GLDC	c.2455A>G	p.Lys819Glu	Del exons 3-20	absent	NA
44	*GLDC*	c.605C>T	p.Ala202Val	c.2665+1G>C	IVS22-1G>C	1
45	*GLDC*	c.1183T>C	p.Phe395Leu	c.2629G>A	p.Glu866*	1
49	*GLDC*	c.1889G>C	p.Arg630Pro	c.1642C>G	p.Leu548Val	NA
70	*GLDC*	c.2315+2T>G	IVS19+2T>G	c.2405C>A	p.Ala802Glu	1
71	*GLDC*	c.605C>T	p.Ala202Val	c.2665+1G>C	IVS22-1G>C	1
73	*GLDC*	c.1738C>G	p.His580Asp	c.1844C>T	p.Pro615Leu	NA

The pathogenic variants causing nonketotic hyperglycinemia in *GLDC* using the sequence NM_000170 ESNT00000321612 and in *AMT* using the sequence NM_000481 ENST00000273588 are provided. If the residual activity has been published, the number of alleles conferring residual activity is listed. NA: not available

**Table 5:** Patient characteristics

Study number	**16**	**17**	**19**	**23**	**28**	**34**	**44**	**45**	**49**	**70**	**71**	**73**
Language	English	Dutch	Dutch	Dutch	English	English	English	French (English)	Dutch	English	English	English
Country	Australia	The Netherlands	The Netherlands	Belgium	USA	USA	Canada	France	Belgium	USA	Canada	USA
Age	10y 8m	16y 11m	10y 4m	7y 7m	14y 5m	15y 8m	18y 9m	11y 10m	12y 0m	12y 2m	21y 0m	12y 7m
Age of diagnosis	13 weeks	5m	17m	5y 2m	12m	12m	Prenatal	3m	3 days	10 days	2m	4 days
Biological sex	Female	Male	Female	Male	Male	Female	Male	Female	Male	Female	Female	Male
Epilepsy	No	No	Yes	Yes	Yes	No	No	No	No	Yes	Yes	Yes
Medication (dose of Benzoate and DMP in mg/Kg/day)	benzoate (275) fluoxetine mirtazapine	benzoate (428)	benzoate (285)	benzoate (294) DMP (4.12) folic acid (co-factor)	benzoate (238) DMP (0.6)	benzoate (250)	benzoate (367) DMP (2.5) clonidine lisdexamp guanfacine fluoxetine lansoprazol	benzoate (450) DMP (7.5) pyridoxal-phosphate haloperidol esomeprazol magnesium carnitine	benzoate	benzoate (258) oxcarbazepine	benzoate (388) DMP(5.2) fluoxetine lansoprazol levonorgestrel, ethinyl estradiol	benzoate (303) DMP (8.7)
Glycine (µmol/L)	700	368	428	292	402	526	240	285	275	360	242	458
IQ or DQ With level of ID	Parent reports “Mild/moderate ID, thinks TIQ around 50”	WPPSI TIQ = 40 Moderate	Parent does not know	NONE	Test unknown TIQ = 43 Moderate	Parent does not know	Stanfort-Binet "Moderate"	NONE	DQ = 60 Mild	NONE	Result unknown to author	NONE

*Abbreviations*: *USA* The United States of America, *y* year, *m* Month, *DMP* Dextromethorphan, *Lisdexamp* Lisdexamphetamine, *Approx* Approximately, *IQ* Intelligence quotient, *DQ* Developmental quotient, *ID* Intellectual Disability, *WPPSI* Wechsler Preschool and Primary Scale of Intelligence, *TIQ* Total IQ-score

### Results of the questionnaires

The salient results of the questionnaires are shown in Table 2 and summarized in Table 6.

**Table 6:** Summary statistics

**Test**		**Mean**	**Standard Deviation**	**Median**	**Minimum**	**Maximum**
DBC	TPS	50.6	17.7	53	15	83
	Disruptive	18.1	8.4	18	3	31
	Self-absorbed	16.7	8.5	17.5	3	30
	Comm. Dist.	6.3	2.3	6.5	2	10
	Anxiety	5.3	2.9	5.5	0	11
	Social relating	3.2	1.7	3	0	6
SCQ	Total score	17	6	16	7	27
	Current	10.9	3.3	10.5	6	15
ABAS	GAC	59.5	8.0	61	47	76
	Conceptual	58.9	7.8	57	49	73
	Practical	61.2	9.2	60	48	82
	Social	68.8	12.3	72.5	54	86
CCC-2	Scales ≥ P95 (n/10)	6.5	2.4	7	3	10
	GCC	123.5	9.6	124	109	140
	Pragm. Score	58.1	5.6	58.5	49	67
	SIDI	-11	4.5	-11.5	-16	-4
MSC	Total	65	22	66	36	109
DCDDaily (i.e.5y)	Participation	49.5	12.5	45	31	68
	Quality	49.8	11.4	52	31	65
M-ABC	TMS	32	17	32	9	60
ADHD	~ DBC (n/9)	6.9	1.4	7	4	9
	~M-ABC (n/5)	3.8	1.1	4	1	5

*Abbreviations*: *TPS* Total Problem Score, *Comm. Dist*. Communication disturbance, *GAC* General Adaptive Composite, *n* Number, *GCC* General Communication Composite, *Pragm. Score* Pragmatic score, *SIDI* Social Interaction Difference Index, *TMS* Total Motor Score

#### Intelligence level and adaptive behavior

According to ICD-10 [24], based on formal assessments provided to us, three children had a total IQ score (TIQ) consistent with moderate ID, and one child with mild ID. Furthermore, for one child parents just indicated mild to moderate ID, whereas four children did not have an intelligence test available. The other parents were not aware of an intelligence test for their child (Table 5).

Adaptive behavior based on the ABAS GAC score revealed very low scores, including very low scores in at least two of the three domains, establishing a quantitative criterion for impairment in adaptive behavior, in keeping with a population with ID. All children achieved very low scores in the Practical and Conceptual domains, whereas the Social domain was a strength, with six of 12 children achieving a low to even a (below) average score. The highest score was in the Social domain for eight children, and the Practical domain for four children.

Of the Dutch speaking children, one child fell below the norm score range for the GAC and for the Conceptual score. A second child fell below the norm for only the Conceptual score. For the remaining children, norm scores for the Conceptual domain had a mean of 59, median 57, range 49 – 73; for the Practical domain a mean score of 61, median 60, range 48 - 82; for the Social domain a mean score of 69, median 73, range 54 – 86; and for the GAC total score the mean was 60, median 61, range 47 - 76. Nine children had a GAC score and 10 a Social domain subscore above the 0.1^the^ percentile, which is discrepantly better than expected for their very low IQ.

#### Language/speech

Three children did not speak more than two-word sentences, and one child failed the validity criterion and was not included for analysis. The eight remaining children had a high GCC (>90^th^ percentile) indicative of a general communication problem (six >99^th^ percentile), affecting most subscales. In contrast to these subscales, only two children scored weak for the Non-verbal Communication Scale and two for the Social Relationships Scale. Seven of eight children had a high pragmatic score (≥90^th^ percentile), indicative of pragmatic language problems. The eighth child with the lower pragmatic score also had the highest ABAS score. Two children used alternative communication tools like signs or communication devices.

#### Motor skills

All questionnaires identified major motor problems.

Scores on the MSC questionnaire indicated that the motor development level of four children was lower than that of children aged 4 y, the lower limit of this questionnaire, and eight children were consistent with children between 4 y 0 m and 5 y 6 m, median 4 y 3 m.

On the DCDDaily-Q, all children took longer to learn ADL (>95^th^ percentile), and the frequency of participation in ADL and quality of performance of ADL was generally low. Children achieved respective scores corresponding to those of children aged 6 y (n=2 and n=1), children aged 5 y (n=2 and n=1), but most of them scored even lower than children aged 5 y (n=5 and n=8). Only two children (#17, #44) achieved participation scores corresponding to children aged 7-8 y (the upper limit of this questionnaire); for one of them, this was also the case for the quality score. One parent mentioned a decrease in motor performance, another parent mentioned good progress in fine motor skills, although not reflected in the measurements. The M-ABC Checklist also identified motor problems, with one child scoring equivalent to 6 y old children, five to 5 y old children, and five scored below that of children 5 y of age, and one child failed scoring for too many "not observed" responses. All parents indicated multiple non-motor factors that affect movement (Part C of M-ABC Checklist), half of them recognize seven or more factors (range 4-11): Distractible (n=12); Hesitant/forgetful, Impulsive and Overactive (n=9); Underestimates own ability (n=8); Disorganized and Lacks persistence (n=7); Timid and anxious (n=6); Overestimates own ability (n=5); Upset by failure (n=4); Passive (n=2); Unable to get pleasure from success (n=1). Parents additionally mentioned chorea (n=2), balance problems (n=3), dystonia and ataxia (n=1 each). Two parents noted variability in motor functioning during the day or over days, depending on emotional state, environment, and how well NKH was controlled.

#### Maladaptive behavior

On the DBC, seven of the 12 children had a TPS in the highest quartile. On the five subscales, four children scored in the highest quartile for the disruptive scale, seven children for the self-absorbed scale, five children on the communication disturbance scale, four children on the anxiety scale (none of which took fluoxetine), whereas no child on the social relating scale. Two children had no maladaptive behavior within the highest quartile across all scales, interestingly both with a mutation in *AMT*.

This problem behavior did not relate to overall functioning. Two of three children with overall better functioning (DBC TPS < P75, SCQ < 15, ABAS GAC > P0.1, Motor > 4y and DBC ADHD symptoms ≤ 6/9), had an abnormal score on the DB Anxiety scale. Further, three children with overall score of very weak performance (ABAS GAC ≤ P0.2, insufficient speech to administer the CCC-2, motor functioning <3y4m), had generally adequate DBC scores.

All parents provided examples of characteristics of the restricted, repetitive behaviors symptom domain for ASD, with nine of 12 parents’ examples fitting at least two characteristics of this symptom domain, which is one of the conditions that supports a DSM-5 diagnosis of ASD [39]. Parents mention several specific fears. For one child, according to the parent, the many hospital interventions in her early childhood may have caused post-traumatic stress disorder.

#### Social functioning and ASD

On the SCQ, the mean score was 17, median 16, range 7-27. Seven children exceeded a score of 15, considered indicative of possible ASD [29, 30]. Of these seven children exceeding the cut-off, when considering their current functioning, only two still meet this cut-off, both with a score of 15. Thus, this data suggests that the children exhibited more autistic behaviors around the age of 4 to 5 y, than they did at the current age of the questionnaire.

On the ABAS, the social domain appeared to be a strength. Six children had a norm score in the low to (low) average range above 70. The highest norm score was for the social domain in two-thirds of children. On the DBC social relations scale, no child scored in the highest quartile. On the CCC2, no child had a high SIDI (>90^th^ percentile), which together with a high GCC would have indicated ASD. Rather, on the contrary, eight subjects had a low SIDI (<10^th^ percentile), while they scored >90^th^ percentile on both the GCC and the pragmatic score. Despite this, only one scored >95^th^ percentile for both Social Relationships and Interests. Three children scored >95^th^ percentile for the Interests Scale, but without an increase in the score for the Social Relationships Scale, and one child scored >95^th^ percentile for Social Relationships Scale while the child had a normal score for the Interests Scale. Three children had normal scores on both scales [31, 32]. Taken together, these results do not indicate a systematic indication for ASD.

#### ADHD

In the DBC questionnaire, 10 parents indicated at least two-thirds of the nine ADHD characteristics present. In the M-ABC Checklist Part C non-motor factors, of the five ADHD related symptoms, five symptoms were present in three children, four in six children, and three in two children. Of all non-motor factors affecting movement, the seven factors present in >50% of children all related to ADHD: Distractible (n=12), Hesitant/forgetful, Impulsive and Overactive (n=9) and Disorganized (n=7). There was no significant correlation between both ADHD related scores (DBC-related ADHD questions and M-ABC Part C ADHD related questions) (Spearman ρ=0.24 p=0.46), reflecting that they evaluated different aspects of this domain. A single child (#17) had less than the 50% of ADHD related symptoms present on M-ABC part C, and had the fewest ADHD symptoms on the DBC, but was not different from other children on the other questionnaires.

### Associations

In an exploratory ANCOVA analysis for factors influencing the TPS on the DBC, which was normally distributed, on a linear model using as variables sex and use of DMP, and as covariates age and last glycine level, only sex and DMP use were significant factors whereas glycine level and age were not. In an optimized model of TPS (F=5.79, p=0.021), predictors sex (F=8.78, p=0.018) and DMP use (F=13.17, p=0.007) were significant. Girls had more problems than boys and children taking DMP had a higher score than those not taking DMP. Similarly, the similar ANCOVA analysis of the ADHD 9 questions score of the DBC had significant predictors of sex (F=9.33, p=0.016, worse in girls) and use of DMP (F=6.41, p=0.035, worse when taking DMP) with the overall model (F=4.17, p=0.047) without a significant interacting term. Similar correlations existed with sex and DMP use on the DBC disrupt score, although the overall model was less significant. Indeed, on the DBC, the TPS significantly correlated with ADHD 9 subscore (R=0.886, p<0.001) and the Disrupt subscore (r=0.899 p<0.001), but not with the DBC Social Relations score. Glycine levels did not correlate with any outcome factor, and there was no difference in any outcome class by glycine level whether in control or not. The single child with both alleles having a variant conferring residual activity (#23) did not fare better than other children. No statistical relationship was found with the ABAS scores.

## Discussion

Parents of children with a good to intermediate attenuated form of NKH very frequently report difficulties to manage behavioral problems in addition to the cognitive dysfunction.

It is striking that objective measures of intelligence and/or adaptive behavior were available in only a minority of the participants, although these measures are less precise and have a wide confidence interval in moderate and severe ID. One would assume that all children with special needs are assessed for educational reasons. In this study, adaptive behavior was rated very low by parents. Interestingly, the adaptive behavior score was higher than expected given the IQ (when available). This was mainly driven by higher scores in the social domain of adaptive behavior. This finding confirms that both IQ and adaptive behavior should be measured when assessing ID [24, 39]. While the social domain was a relative strength, communication skills were weak on the CCC-2 scores. In particular, the language pragmatics scores, which reflect the ability to use available language in communication, were weak. Subscales of the CCC-2 suggest relatively stronger nonverbal communication.

These children experience obvious difficulties in motor development. The overall motor development of the participants was at or below the level of 5 year old children. This development level is lower than the range of adaptive behavior levels or IQ's (when available). This is concerning since over time poor motor development may negatively impact adaptive behavior. Parents further indicate several non-motor factors that may impact their child's motor skills. The frequent presence of neurological motor problem such as chorea may also contribute to poor motor skills.

Autism has been a suggested concern in attenuated NKH. In the SCQ version Life Course, seven of the twelve children obtained a score that is an indication of possible ASD at an earlier age [31, 32]. However, this was not confirmed by the SCQ when assessing current functioning, where only two children scored near the cutoff for the ASD-range. Other questionnaires (CCC-2, ABAS Social domain, DBC Social relations scale) which focused on current functioning, confirmed these results. Clear evidence for autism was not identified for the children in this study.

Ten of 12 parents reported an elevated score on at least one of the DBC scoring areas. This indicates that the participants have maladaptive behavior in at least one domain of functioning. In the subscale Disruptive, most high-scored items related to ADHD-symptoms, while the antisocial behavior related items scored low. Items of the M-ABC Part C questionnaire corresponding with ADHD subdomains also scored high (>50% of the 5 subdomains) in 11 children. Both findings suggest that ADHD is present in many participants, as ten of the 12 children scored on both the ADHD-symptoms of the DBC and the ADHD-subdomains of the M-ABC, though this diagnosis is difficult to delineate in children with moderate to severe ID. Given the frequent ADHD symptoms, it is remarkable that only one child in this study was treated with a psychostimulant (lisdexamphetamine), which is normally the first-line treatment for ADHD. Psychostimulants have been shown to be effective in children with ID [37]. In the experience of the co-author J. Van Hove, a psychostimulant is usually tried but found to be ineffective. In this study, however, no systematic inquiry was made into the history of already tried medications, nor of their effect or lack thereof. Treatment experience could certainly be an area of further research.

**Fig. 1:**
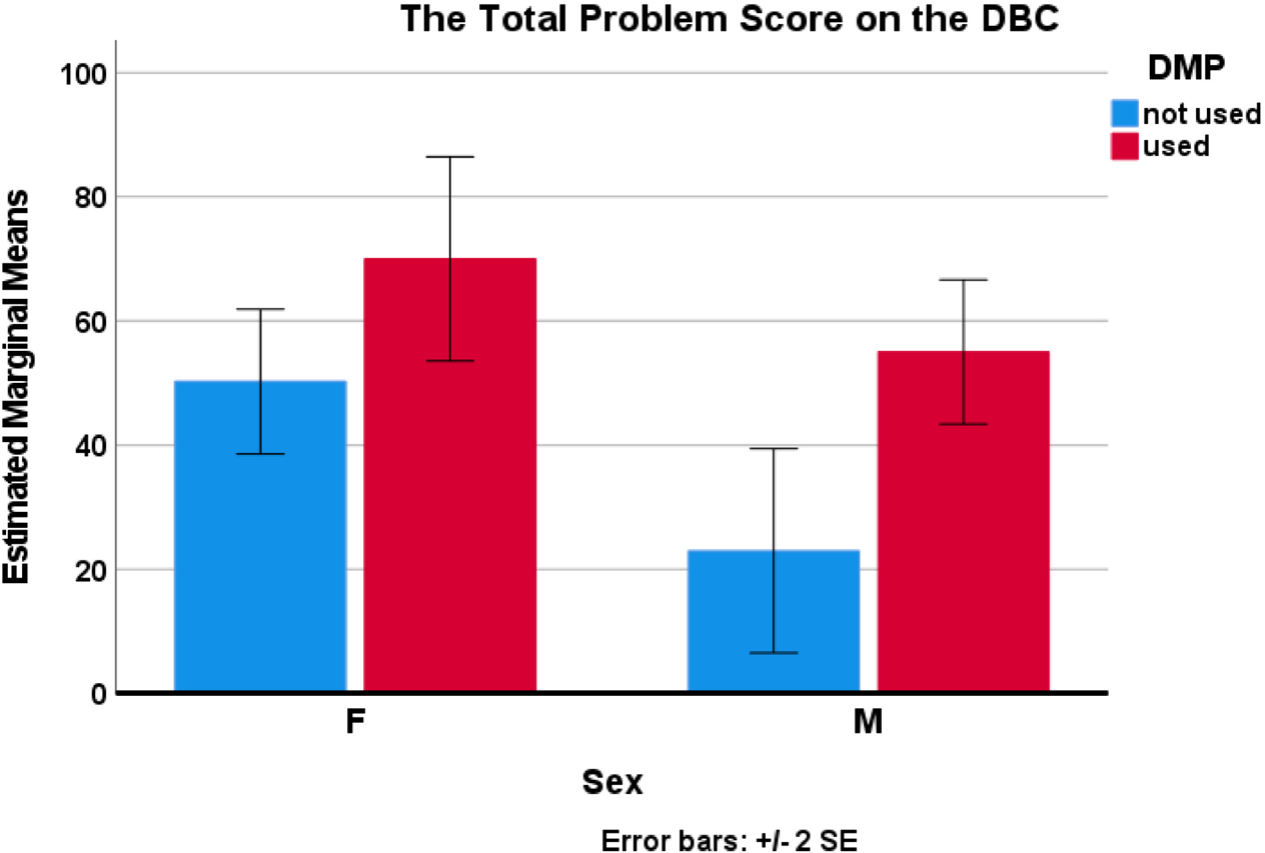
Predictive factors of the Total Problems Score (TPS) on the DBC. In an exploratory analysis of covariance (ANCOVA) for factors influencing the TPS on the DBC, which was normally distributed, and using as variables sex, use of DMP, and as covariates age and last glycine level, only sex (F=7.28, p=0.036) and DMP use (F=14.91, *p*=0.008) were significant factors whereas glycine level and age were not. In a optimized model of TPS (F=5.79, *p*=0.021), predictors sex (F=8.78, *p*=0.018) and DMP use (F=13.17, *p*=0.007) were significant

**Fig. 2:**
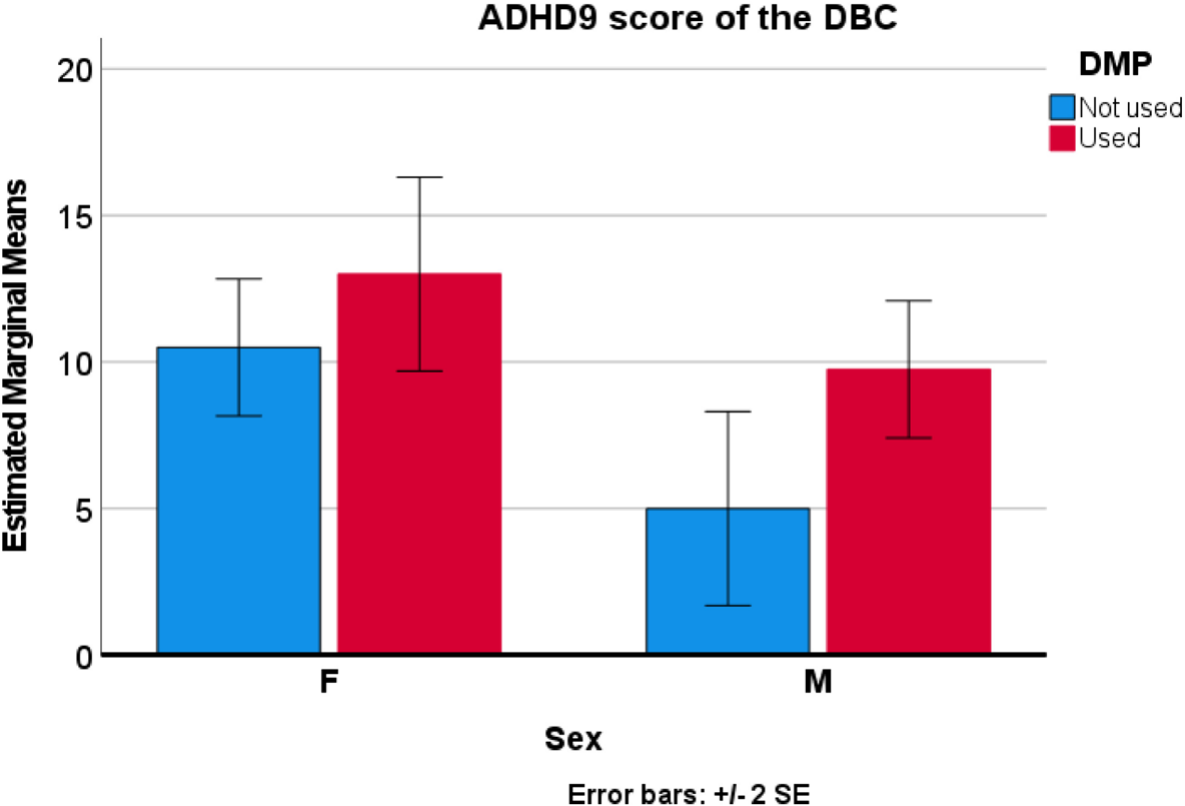
Predictive factors of the Attention Deficit Hyperactivity related question score of the DBC. Similarly, the ANCOVA analysis of the ADHD 9 questions score of the DBC had significant predictors of sex (F=9.33, *p*=0.016) and use of DMP (F=6.41, *p*=0.035) with the overall model (F=4.17, *p*=0.047) without a significant interacting term. The F-test for heteroskedasticity and the Levene’s test for error variances did not show significant factors

The ANCOVA shows a significant association between both DMP use and biological sex on one hand and the total problem score on the DBC (Fig. 1) and the ADHD symptoms within the DBC (Fig. 2) on the other. Higher TPS and more ADHD traits are seen in girls and with DMP use. In contrast, in the general population ADHD is more frequent in boys. A previous study of NKH had suggested a worse outcome in girls [5], but subsequent studies did not confirm this [3, 4]. This observational study cannot discern causation, whether DMP use increases disruptive behavioral problems and ADHD-symptoms, or that children, particularly girls, with a high level of ADHD-symptoms more frequently receive DMP-treatment. This finding should be confirmed on a follow up study to rule out a coincidental finding. Further, a randomized controlled clinical trial would be needed to assess cause and effect. DMP has complex neurochemical effects affecting multiple receptors in addition to the NMDA-type glutamatergic receptors, and its effect can be manifold [41, 42]. There have never been controlled studies on the use of DMP in NKH. The indication for DMP treatment had been as part of standard NKH treatment per published guidelines [2]. This study should make clinicians reconsider the benefit of DMP when presented with disruptive behaviors in children with attenuated NKH. Surprisingly, no influence of (excessive) glycine plasma concentration was seen on maladaptive behavior. A single recent glycine level, which could be obtained in this study, may not reflect the overall glycine burden over time. Similarly, an obvious relation with genetics was not evident. The single child with two residual activity conferring alleles did not fare better than other children with only a single residual activity conferring allele. Associations with DMP dosing or with other drugs could not be evaluated given the heterogeneity and limitations of this study setup.

### Strengths and limitations

The study of an ultrarare condition poses specific challenges. For instance, while we tried to limit recruitment bias by establishing objective inclusion criteria, some bias is inevitable when recruiting through parents’ associations. The study design utilized a questionnaire survey, whereby the participants’ phenotype is constructed through their parents’ observations. A standardized clinical assessment of all children would be preferable, but logistically complex given the geographic dispersion of this ultra-rare condition. Given the extreme rarity of the small subpopulation of this ultra-rare disorder, twelve participants, although few, can still be considered a sizeable number. This represents the only study in which a structured evaluation occurred systematically for all children, focusing primarily on behavioral problems. At the phenotypic level for behavioral problems, only case series or literature reviews had previously been published [5–10]. This group is not large enough to meet all statistical requirements, but is sufficient given the exploratory nature of the study, which primarily consisted of descriptive statistics. It still proved sufficient to enable an initial analysis of predictive factors.

With the exception of the SCQ [29, 30], the reports on the questionnaires are snapshots. Therefore, this study could not reflect the evolution of certain behaviors throughout the development of these children. It proved difficult to find appropriate questionnaires to include the wide age range of the participants, and that could be applied to all surveyed topics. The available questionnaires only addressed younger children, or questionnaires for older children differed too far from the living situation of these intellectually disabled children. We applied as much as possible questionnaires related to the expected range of developmental ages. Like many studies on the behavioral phenotype in groups with a moderate or severe ID, the lack of adequate instruments and norms is a major limitation. Especially in the field of ADHD, this is a shortcoming. Psychometric validation studies on a general population of the proxy instrument, which we compiled based on ADHD related items from the DBC and the M-ABC-2, would be desirable. Additional use of the Aberrant Behavior Checklist (ABC) could potentially add value in further research as this questionnaire seems to capture ADHD characteristics somewhat better than the DBC [37, 38]. The DBC appeared to capture the disruptive behavior and can be an applicable outcome measure to evaluate therapeutic approaches for the behavioral problems that parents report as most disturbing. As none of the standard instruments for assessing child development cover the full range of abnormal behaviors we see in these children, the possibility of constructing a formal instrument for children with NKH should be explored in the future. The Sanfilippo Behavior Rating Scale for children with Mucopolysaccharidosis Type IIIA can be an analogous example of such a disease specific behavioral rating scale [43].

Multiple therapeutic options to address behavior have been proposed ranging from Applied Behavior Analysis (ABA) therapy [2], neuropsychiatric interventions including stimulants or atypical neuroleptics [9], various drugs interfering with NMDA receptor functioning, and even metabolite substitution such as D-serine treatment [44], but any systematic study or even a review of existing experience has been lacking. This study provides a baseline from which to develop such interventional studies. In such studies, the DBC could provide outcome markers for such interventions, including on the effect of DMP.

## Conclusion

Children with the good to intermediate form of attenuated NKH frequently experience problems in their development and behavior. They function at mild to severe ID levels. Their speech/language development is very weak, and their motor development is often even weaker relative to their intellectual functioning. Their adaptive behavior on the other hand, although weak, often looks stronger than their intellectual functioning. There are a few arguments for ASD, however, social domains are a strength in their profile.

Maladaptive behaviors are frequent in particular in the ADHD domain, though only one child in the study received psychostimulants. Girls scored higher on maladaptive behaviors, as did children treated with DMP. Further research into possible causality of this latter finding is recommended. The DBC provided a good measure to capture and study these behavioral problems, including in the context of interventional studies. Surprisingly, no influence of recent plasma glycine concentration was found. We hope that these insights can contribute to better treatment and support for these children and their parents.

## Data Availability

To safeguard privacy given the low number of participants per country, the individual data and materials that support the findings of this study are available upon reasonable request from the authors.

## Abbreviations

NKH: Non-ketotic hyperglycinemia
GCE: Glycine cleavage enzyme
DQ: Developmental quotient
NMDA: N-methyl-D-aspartate
DMP: Dextromethorphan
ID: Intellectual Disability
REDCap: Research Electronic Data Capture
IQ: Intelligence quotient
ICD: International Classification of Diseases
DBC-P: Developmental Behavior Checklist parent form
TPS: Total Problem Score
ABAS: Adaptive Behavior Assessment System
GAC: General Adaptive Composite
SD: Standard deviation
SCQ: Social Communication Questionnaire
ASD: Autism Spectrum Disorder
CCC-2: Children's Communication Checklist
GCC: General Communication Composite
SIDI: Social Interaction Difference Index
MSC: Motor Skill Checklist
DCDDaily-Q: Developmental Coordination Disorder Daily Questionnaire
M-ABC-2-Checklist: The checklist of the movement assessment battery for children
ADL: Activities of Daily Living
ADHD: Attention Deficit Hyperactivity Disorder
TIQ: Total IQ-score
